# Hemodynamic Effects of Anesthetics, Sedatives, and Analgesics in the CICU: Clinical Decision Guidance

**DOI:** 10.1016/j.jacadv.2026.103017

**Published:** 2026-08-26

**Authors:** Zaid N. Safiullah, Willard N. Applefeld, Willy Li, Jeffrey B. Washam, Sylvie Hall, Carlos L. Alviar, Charles Natanson, Michael A. Solomon, American College of Cardiology Critical Care Cardiology Section

**Affiliations:** a Critical Care Medicine Department, National Institutes of Health Clinical Center, Bethesda, Maryland, USA; b Division of Cardiovascular Medicine, Department of Medicine, Duke University School of Medicine, Durham, North Carolina, USA; c Department of Pharmacy, Clinical Center, National Institutes of Health, Bethesda, Maryland, USA; d The Leon H. Charney Division of Cardiovascular Medicine, New York University Langone Medicine Center, New York, New York, USA; e Cardiac Intensive Care Unit and Pharmacy Department, Bellevue Hospital Center, New York, Health and Hospital Corporation, New York, New York, USA; f Critical Care Medicine and Pulmonary Branch, National Heart, Lung, and Blood Institute of the National Institutes of Health, Bethesda, Maryland, USA; g Cardiovascular Branch, National Heart, Lung, and Blood Institute of the National Institutes of Health, Bethesda, Maryland, USA

**Keywords:** CICU, CICU pathophysiology, hemodynamics, sedation and analgesia

## Abstract

Medications used to provide comfort in the cardiovascular intensive care unit exert complex cardiovascular and respiratory effects, necessitating careful selection based on risk-benefit assessments. Hemodynamic effects include changes in vascular tone which can alter preload and afterload, along with chronotropic and inotropic effects which may cause tachyarrhythmias or bradyarrhythmias, affect contractility and lusitropy, and compromise cardiac output and myocardial oxygen consumption. Drug-induced respiratory depression can also be limiting. This review synthesizes evidence on the cardiovascular and respiratory impact of anesthetics, sedatives, and analgesics and proposes a clinical decision framework tailored to pathophysiology. It is essential to recognize that the selection of drugs based on their properties and the patient’s profile and hemodynamic/respiratory reserve provides only an empiric first step. Given varied physiology and disease heterogeneity and high comorbidity burden of cardiovascular intensive care unit patients, it is paramount to start with individualized conservatively dose-adjusted strategies followed by frequent assessment and tailoring of regimens as appropriate.

## Highlights

• In the CICU, despite limited evidence, there is a need to guide anesthetic, sedative, and analgesic selection based on disease pathophysiology as these agents exert divergent hemodynamic effects, necessitating a tailored approach to selection.
• A clinical decision framework is proposed to optimize anesthesia, sedation, and analgesia while mitigating adverse hemodynamic consequences.
• Future research should include CICU-based trials with usual care controls to establish CICU-specific guidelines for anesthesia, sedation, and analgesia strategies.

The cardiovascular intensive care unit (CICU) presents unique anesthetic/sedation and analgesic challenges in managing critically ill patients with cardiovascular disease, many of whom suffer from acute coronary syndromes, heart failure (HF), respiratory diseases, cardiogenic shock (CS), advanced valvular disease, and arrhythmias, all of which may alter hemodynamic reserve and cardiac and respiratory physiology.^1^ About 21% to 24% of CICU patients require invasive mechanical ventilation, with the concomitant need for analgosedation to ensure comfort, improve work of breathing and gas exchange, and optimize hemodynamics.^2^ Other indications for sedation and analgesia in the CICU include management of agitation and facilitation of invasive procedures. Although the incidence of sedation in the CICU is not known, approximately 85% of critically ill patients require sedation, underscoring the importance for the CICU clinician to master sedation and analgesic agent selection in the critically ill cardiac patient.^3^ This narrative review synthesizes current evidence regarding the cardiovascular and respiratory effects of commonly used anesthetic/sedative and analgesic agents, with an emphasis on the pathophysiological challenges presented by CICU patients. By integrating both clinical trial data (Table 1) and expert opinion, we propose a decision framework designed to guide clinicians in selecting agents that maximize efficacy while minimizing adverse hemodynamic and respiratory consequences.

**Table 1:** Summary of Anesthetic and Sedative Clinical Trials: Primary Outcomes From Comparative Clinical Trials of Anesthetic and Sedative Agents

Trial Name	ICU Population	Sample Size	Intervention(s)	Primary Outcome
MIDEX^55^	Mechanically ventilated, critically ill medical/surgical adults requiring sedation >24 h	500 patients, midazolam n = 251, dexmedetomidine n = 249	Dexmedetomidine vs Midazolam	Time at target sedation and duration of mechanical ventilation. Dexmedetomidine was associated with a shorter ventilation duration, but a higher risk of hypotension and bradycardia compared with midazolam
PRODEX^55^	Mechanically ventilated, critically ill medical/surgical adults requiring sedation >24 h	498 patients, propofol n = 247, dexmedetomidine n = 251	Dexmedetomidine vs Propofol	Time at target sedation and duration of mechanical ventilation. Dexmedetomidine was comparable to propofol for the duration for primary endpoints, as well as hypotension and bradycardia
SPICE III^56^	Mechanically ventilated critically ill medical/surgical adults requiring sedation ≥24 h	4,000 patients, dexmedetomidine n = 2,002, usual care (either propofol or midazolam) n = 1,998	Dexmedetomidine vs Propofol or Midazolam or other sedative	90-D mortality. No difference in mortality between dexmedetomidine and usual care groups at 90 d. Significantly more hypotension and bradycardia in the dexmedetomidine group
KEEP-PACE^57^	Mechanically ventilated, critically ill medical/surgical and transplant/oncologic adults undergoing emergency endotracheal intubation	152 patients, ketamine/propofol n = 79, etomidate n = 73	Ketamine/Propofol admixture (“ketofol”) vs Etomidate for intubation	Change in MAP from baseline to 5 min post drug administration No difference in postintubation MAP between ketofol and etomidate groups. Etomidate was associated with more adrenal suppression.
MENDS2^58^	Mechanically ventilated, critically ill medical/surgical patients with known or suspected septic shock	422 patients, dexmedetomidine n = 214, propofol n = 208	Dexmedetomidine vs Propofol	Days alive without delirium or coma within a 14-d intervention period. No difference between dexmedetomidine and propofol in primary endpoint, as well as 90-day mortality, and ventilator-free days

ICU = intensive care unit; KEEP-PACE = Ketamine for Early Endotracheal Intubation/Procedural Comfort and Hemodynamic Stability Evaluation; MAP = mean arterial pressure; MENDS2 = Maximizing Efficacy of Targeted Sedation and Reducing Neurological Dysfunction 2; MIDEX = Midazolam vs Dexmedetomidine for Sedation of ICU Patients; PRODEX = Propofol vs Dexmedetomidine for Sedation of ICU Patients; SPICE III = Sedation Practice in Intensive Care Evaluation III.

Pharmacokinetic properties of drugs such as metabolism and elimination can be altered in the setting of end-organ dysfunction, particularly in shock states characterized by multisystem dysfunction, presenting an additional challenge in caring for critically ill patients. Many agents used to maintain amnesia, comfort, and safety for the patient can negatively impact hemodynamic parameters and respiratory status, making careful selection vital to optimize overall stability.

Agents used for anesthesia, sedation, and analgesia can modulate the level of awareness and pain perception. Anesthetics induce a reversible anterograde amnesia, analgesia, and muscle relaxation, often necessitating ventilatory and hemodynamic support. Sedatives can have effects ranging from conscious sedation to coma by decreasing the level of consciousness in a dose-dependent fashion, with deeper levels frequently impairing respiratory drive and cardiovascular function.^4^ Analgesics decrease the perception of pain or discomfort and can produce states of euphoria; however, doses that are adequate to produce elimination of any pain perception typically also carry the risk of respiratory depression and hypotension. The agents discussed in this review influence these domains to varying degrees^4^^,^^5^; hence, understanding their effects in the context of patients’ cardiovascular physiology, loading conditions, myocardial function, and individual hemodynamic and respiratory reserve is pivotal.

Management of anesthesia, sedation, and analgesia in the CICU benefits from an in-depth understanding of the hemodynamic and respiratory effects of these agents in relationship to the pathophysiology of the cardiovascular and respiratory diseases seen in the CICU. These patients may experience various shock states—including cardiogenic, vasodilatory, or mixed shock—each characterized by distinctive alterations in preload, afterload (systemic vascular resistance [SVR]), cardiac output (CO), myocardial contractility, and respiratory drive. Similarly, CICU patients have characteristics that distinguish them from general ICU populations, including the use of mechanical circulatory support devices and a heightened need to optimize patient-ventilator synchrony—minimizing intrathoracic pressure swings to promote both hemodynamic stability and lung-protective ventilation.

## Hemodynamics of shock states

Shock phenotypes typically managed in the CICU tend to be broader than those managed in other ICU settings. Understanding the various hemodynamic aspects of these phenotypes is important prior to administering agents that have marked hemodynamic effects.

### Cardiogenic shock

CS is a complex heterogenous clinical syndrome where ineffective CO leads to critical end-organ hypoperfusion.^6^^,^^7^ It has a prevalence of 65% in CICU shock patients and carries an overall mortality rate of 40% to 50%^6^^,^^8^ The Society for Cardiovascular Angiography and Intervention (SCAI) categorizes CS into 5 dynamic severity stages (A-E) based on hemodynamic, metabolic, and patient treatment parameters.^9^ Increasing SCAI stage is associated with increasing chance of mortality.^9^ In addition, CS is phenotyped by precipitating etiologies, for example, acute myocardial infarction (AMI), HF, secondary from a nonmyocardial cardiac cause, or postcardiotomy, each associated with varying rates of mortality.^8^^,^^10^ Specific hemodynamic derangements portend worse prognosis.^7^^,^^11^ In these patients, a nuanced understanding of preload and afterload is critical to support myocardial function without imposing an excessive oxygen demand or aggravating ventricular workload.^12^^,^^13^ Advanced monitoring modalities and diagnostic tools (eg, pulmonary artery catheterization, intra-arterial lines, and echocardiography) are often essential to guide hemodynamic support prior to and during the administration of anesthetics, sedatives, and analgesics.

### Vasodilatory shock

Vasodilatory shock, for example, septic shock, is frequently encountered in the CICU with a prevalence of 12% among all patients presenting with shock.^8^ The sepsis cohort of CICU patients tend to be older with more chronic comorbidity and have fewer acute organ failures than their counterparts in noncardiac ICUs, albeit with a similar rate of mortality.^14^ Septic shock, by contrast to CS, is typified by a precipitous drop in SVR.^15^ It is generally associated with an increase in CO, likely due to decreased afterload, increased sympathetic drive and stress response, exogenous catecholamines, and cardiac compensation.^16^^,^^17^ The majority of these patients may develop systolic dysfunction and have uncompensated myocardial depression.^16^, ^17^, ^18^

### Mixed shock

Mixed shock is a syndrome that can be defined as primary, cardiogenic-vasodilatory, or vasodilatory-cardiogenic^18^ and has a prevalence of 20% in the modern CICU. A low SVR is common in mixed shock and can be caused by sterile vasoplegia or sepsis.^19^^,^^20^ Mixed shock states have a similar in-hospital mortality rate to CS, ranging from 23% to 55%,^20^ yet present an even greater management challenge.^18^ In these scenarios, clinicians must balance competing physiologies to optimize myocardial performance. Early and frequent hemodynamic assessment has the potential to allow for optimization of cardiac loading conditions and vasoactive support, especially in complex patients with underlying severe cardiovascular comorbidities requiring anesthesia, sedation, and analgesia.^15^^,^^21^^,^^22^

### Cardiogenic shock with right ventricular failure

CS with predominant right ventricular (RV) failure is also common in the CICU with a prevalence of 25% to 60% of patients with acute pulmonary embolism and 7% of patients with AMI-CS; it is associated with increased mortality compared to isolated left ventricular (LV) failure.^23^, ^24^, ^25^ Shock with an RV failure phenotype requires hemodynamic management considerations beyond those for shock from isolated LV failure. In RV failure, increased RV afterload and RV-pulmonary artery uncoupling reduce RV stroke volume and promote RV dilation and leftward interventricular septal displacement, limiting left heart filling. This process diminishes CO and lowers mean arterial pressure (MAP) and right heart coronary perfusion pressure, reducing RV oxygen supply and worsening ischemia to the right heart which results in an “RV death spiral”.^26^ Management strategies include optimizing preload, reducing RV afterload, enhancing inotropy, and maintaining RV perfusion. As in other shock states, early and frequent hemodynamic monitoring is essential both before initiation and during the administration of anesthetics, sedatives, and analgesics.^26^

## Endotracheal intubation in critically ill cardiac patients

**Figure 1:**
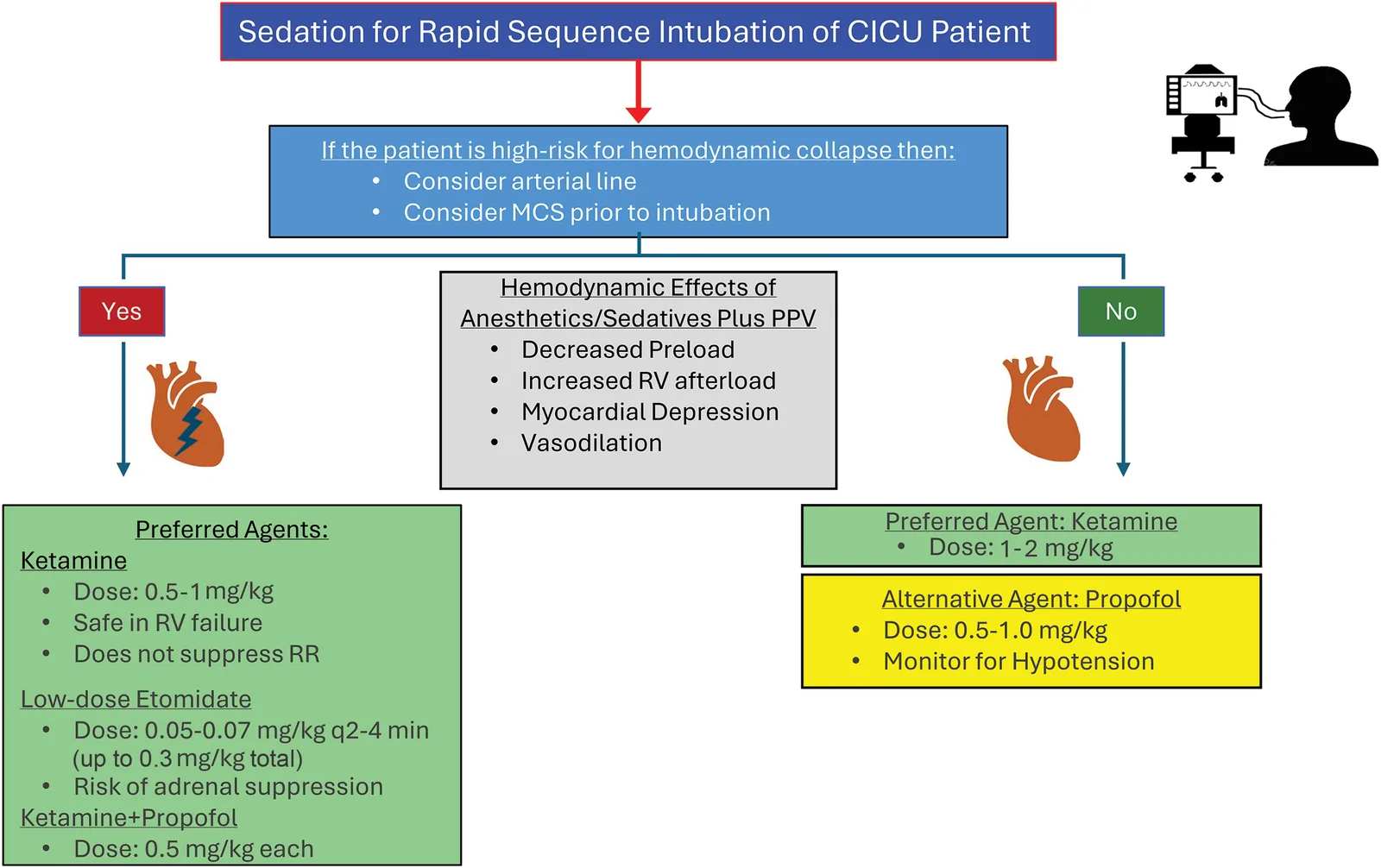
**Anesthetic/Sedative Selection Algorithm for Rapid Sequence Intubation** Agent-selection framework based on the risk of hemodynamic collapse. MCS = mechanical circulatory support; PPV = positive pressure ventilation.

Endotracheal intubation (ETI) of critically ill patients is associated with peri-intubation complications, such as hemodynamic instability and cardiac arrest, with prevalence of 42.6% and 3.1%, respectively, in a large prospective cohort.^27^ ETI poses additional risks in CICU patients. As a general principle, except for some patients intubated for periprocedural intubation or airway protection, the majority of patients in the CICU requiring emergent intubation have an elevated sympathetic response designed to increase heart rate (HR) and vascular tone to compensate for poor cardiac function, which in turn compromises their hemodynamic reserve. The transition to positive pressure ventilation can increase RV afterload and dramatically decrease RV and LV preload. The use of induction agents (anesthetic/sedative medications) during ETI may compound this effect by inducing vasodilation or myocardial depression, precipitating hypotension, and causing potential hemodynamic collapse upon transition to positive pressure ventilation.^28^ Furthermore, at the time of intubation, many patients have concomitant hypoxia and hypercapnia, with associated increased work of breathing, which can worsen acidosis, both metabolic from diaphragmatic muscle use and lactate production and respiratory from impaired ventilation.^29^ This lower pH can further depress systemic vasoactive tone, adding to the already present hemodynamic instability (Figure 1).

Anesthetics, sedatives, and analgesics used in shock states can also suppress the stress response and cause severe hypotension. Stimulation during laryngoscopy can cause tachycardia, bradycardia, or hypertension. If time permits, intubations should include preparation for cardiovascular complications including adequate venous access, vasopressor availability, and an arterial line for monitoring—which can be critical to facilitate rapid treatment in the case of cardiovascular collapse during the peri-intubation period. In RV failure or shock, it is important to maintain systolic blood pressure greater than the mean pulmonary artery pressure to mitigate RV ischemia.^30^ In selected cases of anticipated difficult airway or high risk of hemodynamic collapse, such as in patients with RV failure or shock, high-risk pulmonary embolism, cardiac tamponade, or severe pulmonary hypertension, awake bronchoscopic intubation may offer a safer alternative.^31^ In addition, if time allows and available, it could be reasonable to consider mechanical circulatory support candidacy prior to intubation for these high-risk patients.

## Overview of hemodynamic and respiratory changes induced by anesthetics, sedatives, and analgesics

Anesthetics, sedatives, and analgesics influence several key hemodynamic and pulmonary parameters including preload (central venous and pulmonary capillary wedge pressures), afterload (wall stress, Es/Ea, pulmonary and SVR), inotropy (ejection fraction, CO, end systolic elastance), lusitropy and chronotropy (HR), respiratory drive, carbon dioxide sensitivity, airway muscle tone, and chest wall compliance. Many of these agents induce hypotension primarily by causing vasodilation—either through direct relaxation of vascular smooth muscle or by attenuating a sympathetic tone.^32^^,^^33^ This reduction in left-sided afterload typically translates into a decrease in MAP, a phenomenon that is particularly pronounced in patients with limited cardiovascular reserve.

In addition, some agents depress myocardial contractility by interfering with intracellular calcium handling, which reduces stroke volume. Without adequate chronotropic compensation, a risk factor in patients with compromised cardiac function, CO is reduced.^32^^,^^34^ Alterations in HR—whether manifesting as bradycardia (due to nodal depression) or reflex tachycardia—can further disrupt the balance between oxygen supply and demand.^32^^,^^35^ Agents that diminish preload through venodilation further compromise CO.^35^^,^^36^ The overall effect is a compounded hypotensive response that necessitates careful titration of anesthetic, sedative, and analgesic drugs in the CICU. Finally, many agents depress respiratory drive by inhibitory neuromodulation of brainstem respiratory pathways and can cause apnea leading to cardiopulmonary collapse.

## Anesthetics/sedatives in the CICU Table 2

### Propofol

Propofol acts as a positive allosteric agonist of gamma-aminobutyric acid (GABA)-A receptor, promoting sedation through enhanced chloride conductance. Hemodynamically, propofol administration can cause a reduction in preload, SVR, and myocardial contractility, as well as bradycardia.^32^^,^^37^^,^^38^ Propofol reduces SVR by directly inhibiting calcium influx in vascular smooth muscle cells.^33^ Its decreased CO and bradycardic effects are mediated though inhibition of the sympathetic nervous system and the baroreflex regulatory mechanism.^32^ This can precipitate significant hypotension in critically ill patients,^39^ with peri-induction hypotension reported in up to 16% to 68% of patients undergoing cardiac surgery.^40^^,^^41^ The negative inotropic effects of propofol are controversial, supported by animal studies using supratherapeutic doses outside of the clinical range.^42^ Furthermore, these possible effects may not be clinically meaningful, a retrospective propensity-matched analysis in CS showed decreased mortality in those sedated with propofol compared to midazolam.^43^ Notably, propofol may be favorable in CICU patients with status epilepticus from hypoxic brain injury after cardiac arrest, or in patients requiring deep sedation to quiesce electrical storm.^44^^,^^45^

**Table 2:** Characteristics of Anesthetics and Sedatives: Agent, Mechanism, Maintenance Dose, Clinical Indication, and Prominent Adverse Side Effects as Displayed

Agent	Mechanism	Maintenance Dose in Hemodynamically Stable Patients	Clinical Indications	Notable Side Effects
Propofol	GABA_A receptor agonist; enhances inhibitory neurotransmission	5-10 μg/kg/min, titrated up to 75-80 μg/kg/min	Induction and maintenance of anesthesia; ICU sedation, especially when rapid recovery is desired	Hypotension, bradycardia, respiratory depression, risk of propofol infusion syndrome
Dexmedetomidine	Selective α_2_-adrenergic receptor agonist; decreases sympathetic outflow via catecholamine inhibition	0.2-1.4 μg/kg/h (titrated based on sedation targets)	Sedation of mechanically ventilated patients; nonintubated sedation; procedural sedation in settings where minimal respiratory depression is needed	Bradycardia, hypotension, dry mouth; relatively minimal respiratory depression
Ketamine	NMDA receptor antagonist; exhibits sympathomimetic effects (increases endogenous catecholamines)	For sedation: 0.3-1.0 mg/kg/h; for induction: 1-2 mg/kg	Sedation and analgesia in critically ill patients, particularly in shock states where hemodynamic support is needed	Emergence phenomena (hallucinations, dysphoria), increased secretions, and potential negative inotropy in catecholamine-depleted patients
Midazolam	Potentiates GABA_A receptor activity, enhancing inhibitory neurotransmission	0.02-0.14 mg/kg/h (titrated based on sedation targets)	ICU sedation, anxiolysis, seizure control; useful for short-term sedation	Hypotension, respiratory depression, prolonged sedation (especially in patients with hepatic or renal dysfunction)
Etomidate	Enhances GABA_A receptor activity	Primarily used for induction (0.2-0.3 mg/kg IV); not typically used as maintenance infusion	Rapid sequence intubation in hemodynamically unstable patients; preferred when minimal cardiovascular effects are desired, though repeated dosing may cause adrenal suppression	Adrenal suppression (with repeated doses), myoclonus; minimal hemodynamic effects compared to other induction agents
Atypical antipsychotics	D2 Dopamine receptor antagonist	Quetiapine 25 mg-200 mg/d Olanzapine 2.5-20 mg/d Aripiprazole 2-15 mg/d	Acute hyperactive delirium Agitation refractory to other sedatives	QTc prolongation and anticholinergic effects
Valproic acid	Acts on NMDA, GABA, dopamine pathways, and sodium channels	IV loading dose (optional): 15-20 mg/kg IV/PO maintenance dose: 250-500 mg q8-12 h, max = 60 mg/kg/d	Acute hyperactive delirium Agitation refractory to other sedatives Seizure management	Hepatotoxicity, thrombocytopenia, hyperammonemia

GABA = gamma-aminobutyric acid; ICU = intensive care unit; IV = intravenous; NMDA = N-methyl-D-aspartate.

In addition, extracardiac complications include dose-dependent respiratory depression, hypertriglyceridemia (up to 15% to 30% of ICU patients),^46^ and propofol infusion syndrome—a rare but potentially fatal complication characterized by metabolic acidosis, rhabdomyolysis, and cardiovascular collapse. The risk of propofol infusion syndrome increases with infusions exceeding 4 mg/kg/h for more than 48 hours and warrants, at minimum, weekly monitoring of serum triglycerides, creatine kinase, acid-base status, and lactate levels during prolonged administration.^47^^,^^48^ For this reason, propofol is best reserved for short-term sedation to avoid accumulation.^44^^,^^45^

*Clinical Pearl: For the CICU patient, propofol is favored for short-term sedation and for its rapid onset and* *rapid offset* *and is preferred to midazolam but carries a risk of significant hypotension, bradycardia, and apnea. It requires careful titration and airway management in patients with underlying cardiovascular disease.*

### Dexmedetomidine

Dexmedetomidine is a selective alpha-2 adrenergic receptor agonist that exerts its effects by reducing catecholamine release through presynaptic alpha-2 receptor activation. This results in sedation and anxiolysis with minimal respiratory depression. Its sympatholytic properties lead to reductions in preload, SVR, and HR, and in those with diastolic dysfunction, it may reduce myocardial contractility.^49^, ^50^, ^51^ The sympatholytic effect caused by dexmedetomidine can precipitate significant bradycardia and hypotension—reported in over 40% of ICU and cardiac surgery patients^52^—and, in rare cases, sinus arrest, compromising hemodynamic stability.

In critically ill surgical patients after a major abdominal surgery, the HR and MAP were lower in those sedated with dexmedetomidine than in those sedated with propofol. However, there was no difference between groups in cardiac index, incidence of bradycardia, and significant hypotension.^50^ A meta-analysis of sixteen randomized controlled trials in critically ill medical and surgical patients illustrated that dexmedetomidine compared to other sedatives reduced ICU length of stay, duration of mechanical ventilation, and the incidence of ICU delirium, although there was a significant increased risk of hypotension and bradycardia.^53^ Lastly, a recent 2025 clinical trial compared dexmedetomidine to propofol in medical/surgical/trauma ICU patients and found no difference in time to extubation but found that dexmedetomidine was associated with more severe bradycardia than propofol.^54^ Taken together, these studies indicate dexmedetomidine could promote faster recovery but may be associated with more hypotension and bradycardia than propofol. However, the comparative effects of dexmedetomidine and propofol have not been studied prospectively in CICU patients; therefore, it is unclear if these findings can be generalized to critically ill patients with cardiovascular disease.

The MIDEX (Dexmedetomidine vs Midazolam for Continuous Sedation in the ICU) trial compared midazolam with dexmedetomidine for sedation in prolonged mechanical ventilation in medical, surgical, and trauma ICU patients (Table 1). Dexmedetomidine was associated with a shorter duration of mechanical ventilation without significant differences in ICU length of stay or mortality compared to midazolam. However, there was a greater risk of hypotension and bradycardia with dexmedetomidine.^55^ Similar to MIDEX, the SPICE (Sedation Practice in Intensive Care Evaluation) III trial, conducted in medical/surgical ICU patients, found increased rates of hypotension, bradycardia, and sinus arrest in the dexmedetomidine arm compared to the arm treated with the usual care sedation.^56^ Although the current evidence suggests that dexmedetomidine is beneficial in reducing the duration of ICU length of stay and mechanical ventilation, it is also associated with more adverse hemodynamic effects and conduction impairment than propofol or midazolam.^55^^,^^59^

*Clinical Pearl: In the CICU patient, dexmedetomidine is associated with more hypotension, bradycardia, and an increased vasopressor/inotrope requirement compared with other sedatives*.^59^ *Therefore, caution is warranted in patients with acute decompensated HF or high-grade conduction disease. Furthermore, the negative chronotropic effects attenuate the compensatory tachycardia that these patients often rely on* *to maintain cardiac output, potentially leading to hemodynamic deterioration.*

### Ketamine

Ketamine, a dissociative anesthetic, is a phencyclidine derivative that acts primarily as an N-methyl-d-aspartate receptor antagonist, inhibiting excitatory neurotransmission in the central nervous system. Unlike other anesthetics/sedatives, ketamine possesses sympathomimetic properties due to its ability to block reuptake of catecholamines, which in turn increases bronchodilation, preload, SVR, HR, and CO.^60^ This effect makes ketamine a more favorable agent for anesthesia/sedation in patients with tenuous shock physiology, right HF, or at risk of bronchospasm.^61^ Furthermore, in children with pulmonary hypertension, ketamine appears to be safe and is not associated with marked hemodynamic changes.^62^, ^63^, ^64^ However, caution is warranted as it can exert direct myocardial depressant effects in catecholamine-depleted states, where the advantage of blocking catecholamine reuptake is mitigated, or with severe LV dysfunction, an important consideration in critically ill patients.^60^^,^^65^^,^^66^ Adverse effects of ketamine include emergence phenomena, which occurs in 10% to 20% of adult patients receiving ketamine for procedural sedation and is characterized by hallucinations, vivid dreams, delirium, and agitation—particularly with higher or repeated dosing.^67^ These reactions may be mitigated by coadministration of benzodiazepines and require close neurological monitoring during recovery.

There are mixed data on ketamine’s hemodynamic effects in ICU patients. In a retrospective analysis, in diverse ICUs, including CICUs, when compared to dexmedetomidine and propofol for continuous sedation, ketamine was associated with less hypotension and bradycardia.^68^ In contrast, a smaller retrospective analysis of ICU patients (n = 68) with a diverse shock physiology showed an increase in vasopressor requirements associated with ketamine compared to midazolam or propofol.^69^ This discrepancy could be due to dose-dependent myocardial depression. Studies that examined the hemodynamic effects of ketamine in mechanically ventilated patients had heterogenous findings. Some showed increased HR and MAP and decreased vasopressor requirements, while others reported hypotension.^60^^,^^70^ In addition, ketamine has been studied as an adjunctive agent to fentanyl and propofol to augment sedation and analgesia. In these studies, it decreased overall analgesia and sedation requirements, leading to longer time at target sedation goals, without increasing the risk of hypotension or bradycardia.^71^ The varying hemodynamic effects of ketamine in the critically ill may depend on the severity and chronicity of the critical illness^60^^,^^72^ as ketamine has the potential to depress myocardial function in chronically ill patients who are catecholamine depleted. Additional prospective studies in critically ill populations comparing ketamine to usual care are needed to clearly elucidate its hemodynamic effects.

*Clinical Pearl: Despite limited CICU patient–specific data, because of its hemodynamic profile and minimal respiratory depression, ketamine is considered a preferred analgesic/sedative agent in select patients with shock physiology, right HF, or at risk of bronchospasm*.^70^ *Careful monitoring of cardiac function, mental status, and emergence reactions* *is essential when using ketamine in the CICU.*

### Midazolam

Midazolam, a short-acting benzodiazepine, exerts its effects by potentiating GABA-A receptor activity, enhancing inhibitory neurotransmission in the central nervous system. This results in sedation, anxiolysis, muscle relaxation, and anticonvulsant effects. Hemodynamically, it reduces preload, SVR, HR, and CO and may alter myocardial contractility. Its rapid onset and short half-life make it a popular choice for procedural sedation in ICU settings. However, in critically ill patients, particularly the elderly, its metabolism can be prolonged because of hepatic or renal dysfunction, leading to drug accumulation, more severe respiratory depression, and unpredictable effects on sedation depth and recovery.^73^ Midazolam provides effective sedation and anxiolysis, particularly in patients requiring short-term mechanical ventilation. Its titratable effect and rapid onset allow for rapid control of agitation or anxiety. In addition, midazolam has anticonvulsant properties. However, midazolam can cause dose-related hypotension in 10% to 20% of ICU patients.^55^^,^^74^ In patients with CS or severe HF, midazolam should be used judiciously to prevent hypotension and respiratory depression, and flumazenil which can reverse these effects, should be available. Furthermore, midazolam has been shown to have increased rates of delirium and mortality, ICU length of stay, mechanical ventilation duration, and marked respiratory depressive effects, compared to other sedatives in mostly medical and surgical ICU patients.^43^^,^^75^ When midazolam is selected for sedation, the unwanted side effects can be mitigated by scheduled bolus dosing with additional as-needed boluses for breakthrough agitation rather than continuous infusions.

*Clinical Pearl: In the CICU patient, midazolam may be considered in specific scenarios requiring short-term sedation, seizure control, or management of sympathetically driven cardiovascular pathologies like electrical storm. It is limited by its respiratory depressive effects and potential for accumulation which may delay neuroprognostication, ICU liberation, and recovery.*

### Etomidate

Etomidate is a nonbarbiturate hypnotic that is a positive allosteric modulator of GABA-A receptors, enhancing inhbioatory neurotransmission in the central nervous system.^76^ This results in potent sedative effects without worsening arrhythmias or reducing preload, SVR, HR, CO, and myocardial contractility.^77^ It also causes dose-dependent inhibition of 11-beta-hydroxylase that impairs cortisol production, leading to adrenal suppression, which has an incidence of 80% in critically ill patients.^78^^,^^79^ Etomidate should not be used for extended sedation, and mostly limited to rapid sequence intubation (RSI) in patients with advanced HF, but may be used more liberally in patients on supplemental steroids. However, even a single dose of etomidate during ETI of critically ill patients has been linked to increased mortality^80^; therefore, it should be used cautiously, and serum cortisol levels should be monitored for 24 to 48 hours following administration in patients with suspected adrenal suppression. Furthermore, it can precipitate a 20% to 30% reduction in cerebral blood flow, causing a reduction in intracranial pressure.^77^^,^^78^^,^^81^

A large retrospective analysis of over 12,000 patients compared its effects to propofol administered on the first day of mechanical ventilation. Etomidate was associated with increased all-cause mortality and reduced odds of 48-hour survival compared to propofol. There was no difference in cardiovascular morbidity between groups.^80^ Similarly, a retrospective analysis of 44,546 mechanically ventilated patients compared the effects of etomidate vs ketamine. Etomidate was associated with increased hospital mortality compared to ketamine that was not attenuated by steroid administration.^82^ However, these studies included a sicker non-CICU population with a higher prevalence of hypotension and vasopressor use in the etomidate groups, meaning that the ostensible association between etomidate and increased mortality may be confounded by severity of critical illness. In addition, a recent clinical trial that compared etomidate vs ketamine for RSI of critically ill patients showed no difference in 28-day in-hospital mortality.^83^

*Clinical Pearl: For the CICU patient, etomidate for RSI induction is not* *the usual* *first-line agent in hemodynamically stable patients because of is adrenal suppressive effects; however, it has a role in the induction process for patients with shock, HF, and malignant arrhythmias where there is no safer alternative (*Figure 1*). One should remain mindful of its adrenal suppression effects and possible mortality risk.*

## Adjunctive sedative agents

Typical and atypical antipsychotics are often used as adjunctive agents to manage refractory agitation and hyperactive delirium in the CICU. Delirium can occur in about 20% of CICU patients.^84^ Typical antipsychotics (eg, haloperidol) are D2 dopamine receptor antagonists, while atypical antipsychotics (eg, quetiapine, olanzapine) additionally antagonize serotonin receptors. Adverse effects of these agents include corrected QT interval (QTc) prolongation, hypotension, and ventricular tachycardia.^85^ There are limited data on the safety and efficacy of antipsychotics in the CICU; however, atypical antipsychotics are generally preferred over typical antipsychotics because of a lower risk of extrapyramidal symptoms.^86^ A small retrospective study of 144 CICU patients showed 14% of patients receiving atypical antipsychotics had significant QTc prolongation and that 2% developed ventricular tachycardia. These adverse effects were more common in those receiving quetiapine than in those receiving olanzapine.^87^ Additional prospective studies are needed to fully elucidate their safety and efficacy in the CICU.

Valproic acid, an anticonvulsant that attenuates N-methyl-d-aspartate pathways and modulates GABA and dopaminergic pathways, has also been trialed in the acute management of hyperactive delirium and agitation. While it has not been associated with hypotension or QTc prolongation like antipsychotics, valproic acid can cause hepatotoxicity, hyperammonemia, and thrombocytopenia, necessitating careful consideration prior to initiation. A small retrospective study in critically ill patients, including CICU patients, found that its administration reduced the need for midazolam and the overall exposure to concomitant sedatives by day 3 of treatment.^88^

*Clinical Pearl: In the CICU patient, while atypical antipsychotics can increase the risk of arrhythmias, their short-term use may be warranted in patients with significant distress from delirium symptoms. In addition, the anticonvulsant valproic acid could be beneficial as an adjunctive agent that may mitigate the need for additional sedatives with adverse hemodynamic effects, but liver function and platelet levels should be* *monitored.*

## Analgesics in the CICU (Table 3)

### Opioids

Opioids like fentanyl and morphine act on mu-opioid receptors in the central nervous system to modulate pain perception. They provide potent analgesia and can be used as monotherapy to provide analgesia or with sedatives for synergistic effects on analgesia and sedation in appropriate patients.^5^ Their use can be limited by systemic effects because of their interaction with peripheral opioid receptors and the autonomic nervous system.^89^ Hemodynamically, opioids can cause bradycardia and decreases in preload, SVR and CO. This is mediated by a direct action on the opioid receptor and indirectly by an induced histamine release.^90^ Fentanyl and hydromorphone are ubiquitously prescribed in ICU practice. Fentanyl exerts modest hemodynamic changes and is associated with less histamine release.^90^^,^^91^ Hydromorphone is longer acting than fentanyl, providing more sustained analgesia. It is associated with more histamine release than fentanyl.^90^^,^^92^ All opioids have the potential to cause decreases in respiratory drive through their activity in the brainstem.^89^ Fentanyl has the potential for tachyphylaxis with continuous infusion and rarely can cause serotonin syndrome. In rapid high doses, it can reduce chest wall compliance, impairing respiratory status. The respiratory suppressive effects can have significant implications when trying to avoid invasive ventilation, as well as when attempting extubation. Although there are several reversal agents available (naloxone, nalmefene), these carry a risk of adverse cardiovascular events, and are best to be rendered unnecessary by careful titration. In addition, opioids may lead to addiction, drug-induced constipation, or ileus, so the use of aggressive bowel regimens is prudent for patients to avoid complications associated with ileus and to avoid potential cardiac events associated with straining and vagal nerve stimulation. Opioids provide effective analgesia and decrease cough, which is important in mechanical ventilation, post–cardiac surgery care, and during invasive procedures. However, their adverse hemodynamic and respiratory effects (Table 3) can be compounded when used in conjunction with anesthetics and sedatives.

**Table 3:** Characteristics of Analgesics: Agent, Mechanism, Maintenance Dose, Clinical Indication, and Prominent Adverse Side Effects as Displayed

Agent	Mechanism	Typical Dose/Administration	Clinical Indications	Notable Side Effects
Opioids (eg, fentanyl)	Mu-opioid receptor agonist; modulates central pain pathways	Fentanyl IV infusion: ∼25-300 μg/h (titrated to effect)	Primary analgesia in critically ill patients; part of sedation protocols in the ICU	Respiratory depression, hypotension, bradycardia, risk of tachyphylaxis
Acetaminophen	Central analgesic and antipyretic; inhibition of cyclooxygenase in the CNS and modulation of descending pain pathways	1 g IV every 6 h (adjust dose based on patient factors)	Adjunct analgesia to reduce opioid requirements; mild-to-moderate pain control	Potential for hypotension (IV), hepatotoxicity in overdose
NSAIDs	Inhibit cyclooxygenase (COX) enzymes, reducing prostaglandin synthesis and thus inflammation and pain	Varies by agent and route; dosing must be individualized (oral or IV)	Adjunct analgesia and anti-inflammatory effects; used to lower opioid consumption	Gastrointestinal bleeding, renal impairment, increased cardiovascular risk, volume overload, platelet dysfunction
Lidocaine	Sodium channel blocker; stabilizes neuronal membranes, providing analgesia; also has antiarrhythmic effects	Initial bolus: 1-1.5 mg/kg Maintenance infusion: 1-2 mg/kg/h	Adjunct analgesia in the ICU; reduction of opioid use; antiarrhythmic effects	CNS toxicity (eg, seizures, tinnitus), cardiac conduction abnormalities, hypotension
Bupivacaine (epidural)	Local anesthetic; blocks sodium channels to inhibit nerve conduction	Initial bolus: 5-15 mL of 0.25%-0.5% solution Maintenance infusion: 0.0625%-0.125% at 6-12 mL/h	Regional analgesia for postoperative pain management	Hypotension (from sympathetic blockade), motor block, potential for local anesthetic systemic toxicity

CNS = central nervous system; ICU = intensive care unit; IV = intravenous; NSAIDs = nonsteroidal anti-inflammatory drugs.

Opioids are first-line agents for analgesia in the ICU.^93^ In mechanically ventilated patients, fentanyl is preferred to morphine for continuous analgesia because it is associated with less histamine release and hypotension,^94^ a lack of accumulation of active metabolites in renal dysfunction, increased ventilator-free days, decreased ICU length of stay, and similar ICU mortality.^95^ The use of opioids in conjunction with nonopioids for analgesia can decrease the overall opioid requirement, perceived pain score, duration of mechanical ventilation, and ICU length of stay.^96^ Notably, there was no difference in hemodynamic instability between opioid and opioid plus nonopioid analgesic groups.

*Clinical Pearl: For the CICU patient, opioids are critical for analgesia and* *can* *augment* *the effects of sedatives,* *but require careful* *dose titration* *and frequent monitoring to provide adequate pain relief without causing respiratory depression and/or hemodynamic instability (*Central Illustration*).*

**Central Illustration:**
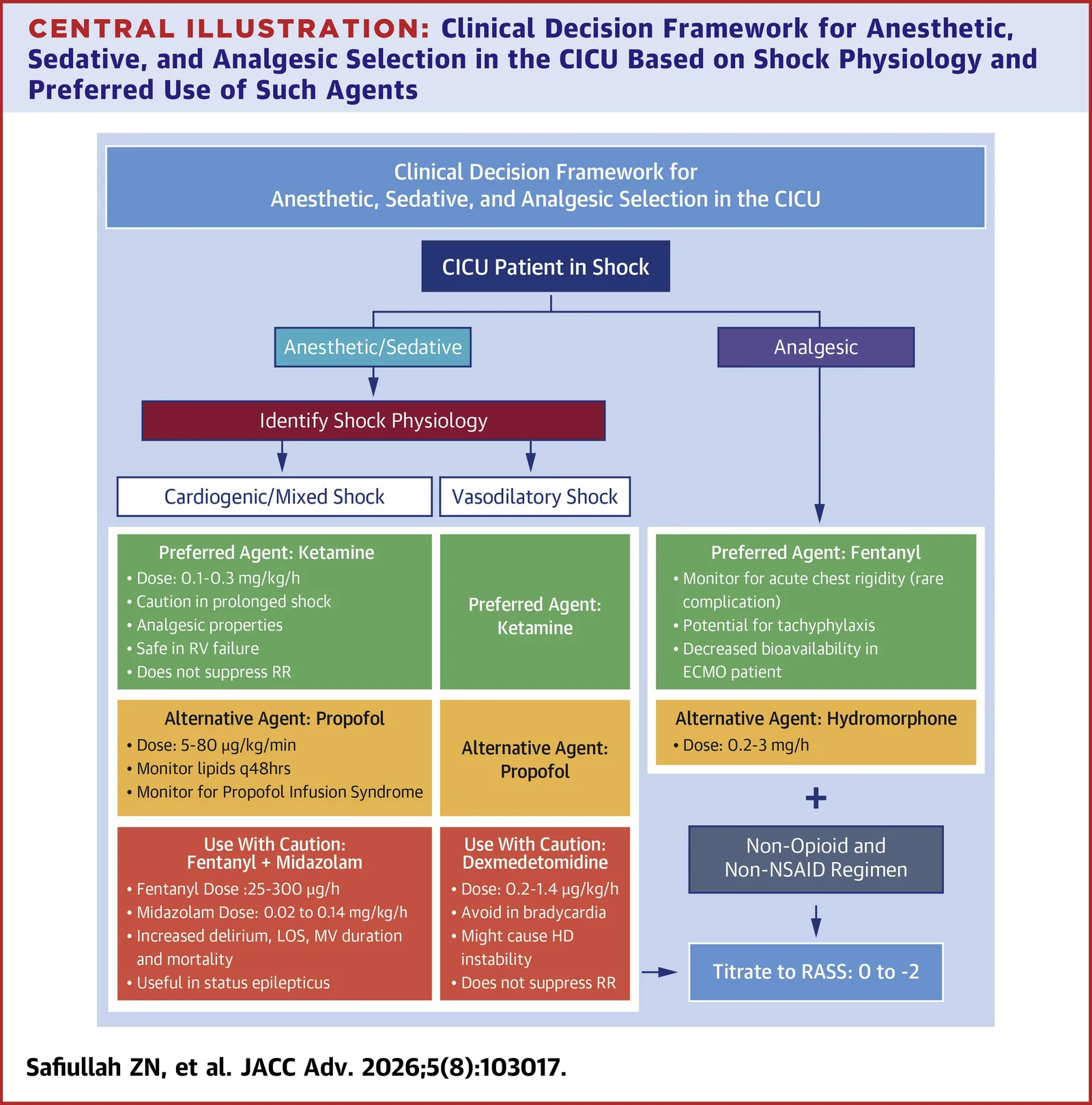
**Clinical Decision Framework for Anesthetic, Sedative, and Analgesic Selection in the CICU Based on Shock Physiology and Preferred Use of Such Agents** CICU = cardiac intensive care unit; ECMO = extracorporeal membrane oxygenation; HD = hemodynamic; LOS = length of stay; MV = mechanical ventilation; NSAID = nonsteroidal anti-inflammatory drugs; RASS = Richmond Agitation Sedation Scale; RR = respiratory rate.

### Nonopioid analgesics

Acetaminophen is a first-line agent for analgesia. Its main analgesic mechanisms are activation of the transient receptor potential vanilloid 1 and cannabinoid 1 receptors in the central nervous system through its active metabolite N-acylphenolamine and central cyclooxygenase inhibition.^97^ Oral acetaminophen has been traditionally considered to be hemodynamically inert; however, regular use can raise systolic blood pressure in hypertensive patients.^98^ Intravenously administered acetaminophen can acutely cause hypotension by decreasing preload and SVR in critically ill patients and those undergoing cardiac surgery.^99^, ^100^, ^101^ In the critically ill, adjuvant use of acetaminophen for analgesia can decrease total opioid requirements.^96^ Adversely, acetaminophen can cause drug-induced liver injury, especially in patients who have had a prolonged period of oxidative stress and may be glutathione depleted, such as those with prolonged ICU stays, chronic decompensated HF, liver disease, or prolonged periods of stress (Table 3).

Nonsteroidal anti-inflammatory drugs (NSAIDs) act by inhibiting cyclooxygenase enzymes, which are essential for prostaglandin synthesis. Prostaglandins play a critical role in pain and inflammation but also regulate renal blood flow and gastrointestinal mucosal integrity.^102^ Hemodynamically, the inhibition of prostaglandins can lead to increases in SVR and increased preload through augmented renal sodium retention, consequently increasing MAP.^103^, ^104^, ^105^ In critically ill patients, NSAIDs as adjuvant analgesia to opioids reduce overall opioid use.^106^ However, they have significant risks including gastrointestinal bleeding, acute kidney injury, HF exacerbation, and platelet dysfunction (Table 3).^104^

Lidocaine is a sodium channel blocking agent that has analgesic, anti-inflammatory, and antiarrhythmic effects.^107^ Hemodynamically, intravenous administration can cause bradycardia and reduced myocardial contractility, leading to decreased CO and hypotension.^108^ Lidocaine infusions are beneficial in critically ill patients by reducing pain scores and opioid requirements. However, it is associated with significant adverse effects, such as neurotoxicity, bradycardia, heart block, and hypotension.^107^ Adverse hemodynamic effects from transdermal application are rare; however, systemic absorption can still occur (Table 3).^109^

*Clinical Pearl: In the CICU patient, acetaminophen is useful to mitigate opioid use but should be cautiously used in those with liver disease, and intravenous administration has the potential to worsen hypotension in those with tenuous hemodynamics. NSAIDs have a limited role in analgesia because of their significant adverse effects of worsening volume overload, gastrointestinal bleeding, kidney injury, platelet inhibition, and increased risk of* *adverse cardiac events and should be used with caution. Intravenous lidocaine administration can have beneficial effects including pain and tachyarrhythmia control but requires* *careful* *monitoring to mitigate adverse effects.*

### Regional and local analgesia

Bupivacaine has a similar mechanism of action to lidocaine but is longer acting. Bupivacaine can be administered by thoracic epidural infusion in combination with an opioid. Epidural anesthesia can precipitate cardiac sympathetic fiber blockade, leading to hypotension from bradycardia, decreased myocardial contractility, and decreased preload and SVR.^110^ In the critically ill patient, thoracic epidural anesthesia can reduce the systemic opioid requirement but may lead to hypotension and prolonged mechanical ventilation (Table 3).^111^^,^^112^ Importantly, placement of an epidural carries the risk of spinal hematoma, and this risk is increased among patients taking antithrombotic agents.^113^ Given the high rate of anticoagulant and antiplatelet use in the CICU population, plans to place an epidural should include discussion with anesthesiology and consultation of existing guidelines to determine appropriate antithrombotic washout period prior to insertion and removal.^114^

*Clinical Pearl: In the CICU patient, epidural anesthesia can be useful in reducing* *systemic opioid requirements* *and myocardial oxygen consumption.*^110^ *However, it must be used cautiously to mitigate adverse hemodynamic effects.*

## Metabolism of anesthetics, sedatives, and analgesics

**Figure 2:**
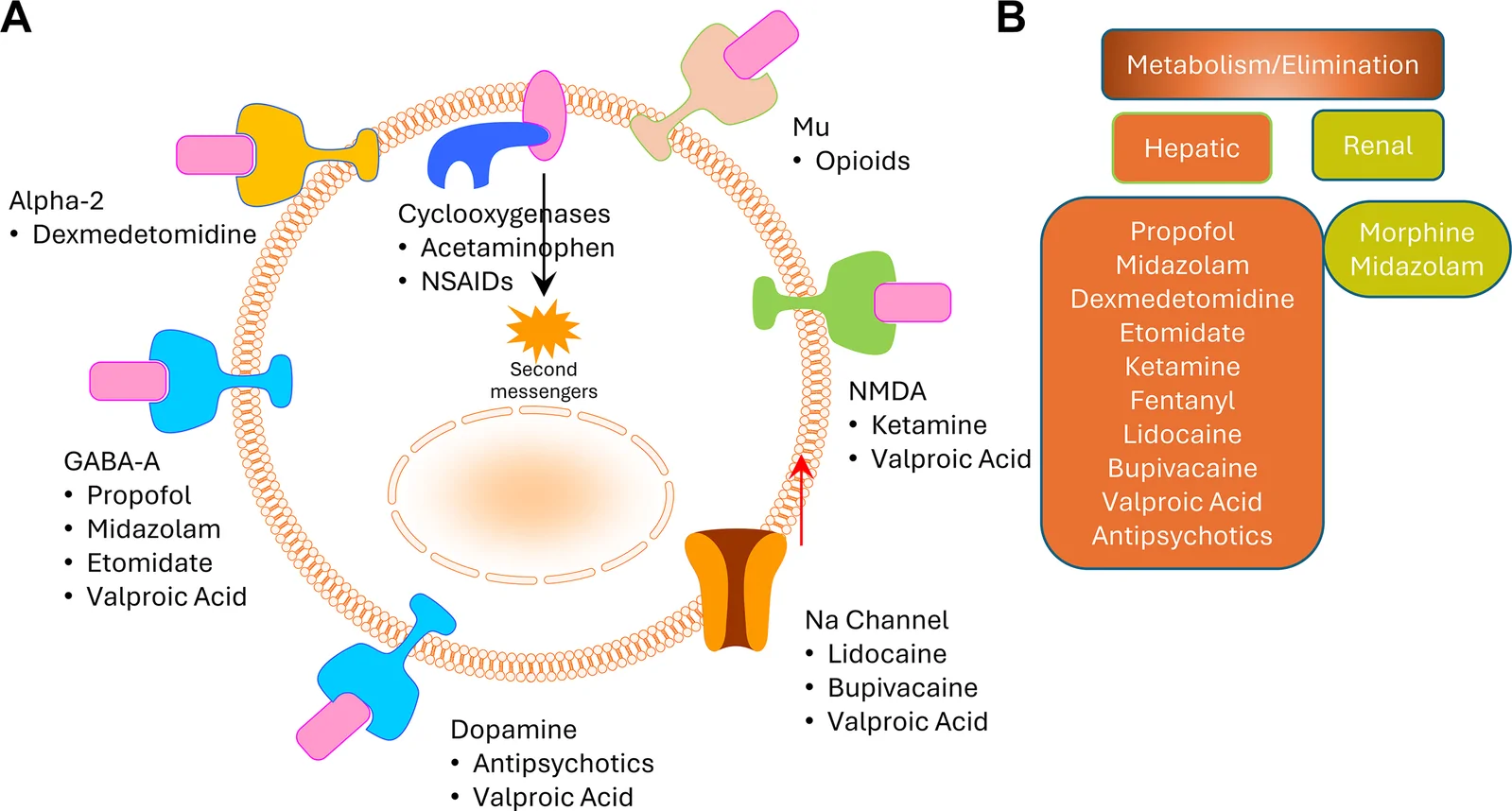
**Mechanisms, Metabolism, and Elimination of Anesthetics, Sedatives, and Analgesics** (A) Drug receptor targets. (B) Metabolism/elimination of agents: most agents are largely metabolized in the liver except for morphine derivatives. Figure created with the Motifolio Biomedical PPT Toolkit (www.motifolio.com). GABA = gamma-aminobutyric acid; NMDA = N-methyl-D-aspartate.

The anesthetics, sedatives, and analgesics discussed are primarily metabolized in the liver, either by cytochrome P450 enzymes or biotransformation, then renally eliminated.^60^^,^^77^^,^^115^^,^^116^ Hepatic and renal dysfunction are common in critical illnesses and can impair the clearance of these agents, increasing the risk of toxicities and drug interactions. Routine hepatic and renal function monitoring and consultation with a critical care or cardiology pharmacist for dose adjustments are vital.^117^ Figure 2 illustrates the mechanisms of the anesthetics, sedatives, and analgesics discussed and categorizes their metabolism.

## Clinical decision framework

There is no clear consensus on an optimal sedation strategy in CS, in part because of its heterogeneity; as such, we recommend an approach tailored to individual patient physiology, comorbidities, and severity of illness.^118^^,^^119^ There are limited data to guide anesthetic/sedative choice in the subtypes of CS, for example, AMI-CS, HF-CS, or secondary CS. Midazolam was previously considered a safer sedative agent than propofol in CS because it was believed to cause less hypotension.^119^ However, a recent observational study in the CICU suggested that sedation with propofol was associated with decreased 30-day mortality, lower exogenous catecholamine doses, and fewer adverse bleeding events compared to those treated with midazolam.^43^ For analgesia, fentanyl is commonly used, but it has not been studied in this specific population. Furthermore, shock-associated hepatic and renal dysfunction can impair the clearance of these agents. Regrettably, there is a paucity of well-designed trials with controls receiving usual care to guide anesthetic, sedative, or analgesic choices in the CICU.

Ketamine has the potential to promote hemodynamic stability based on the catecholamine status of a patient. Ketamine increases CO through a sympathomimetic mechanism.^60^ However, clinical outcomes with ketamine are inconsistent, and some patients may experience hypotension. These differential hemodynamic effects could be related to the degree of preadministration catecholamine depletion.^72^ We speculate that patients with less-severe CS (SCAI Stage B) may benefit more from ketamine administration than their SCAI Stage C and D counterparts, who may be more catecholamine deplete. Yet, there are limited data to support the use of specific agents for different shock SCAI stages. The effect of etomidate in RSI is theoretically hemodynamically stable. However, it has been associated with increased mortality, and in some studies, it was associated with a similar incidence of hypotension after ETI compared to other anesthetics/sedatives.^81^ Thus, for the most part, ketamine may be preferred. However, in patients with reduced sympathetic reserve, such as those with advanced or chronic HF, etomidate may be a reasonable alternative to ketamine.

We suggest a sedation trial with low doses of ketamine for patients with CS in whom an increase in catecholamine levels is not contraindicated (Central Illustration, Table 2). Whereas the hemodynamic outcomes with ketamine for anesthesia/sedation are conflicting, the associated hypotension with propofol, dexmedetomidine, midazolam, and fentanyl is more consistent. Propofol can be used as an alternative agent, particularly if only short-term therapy may be needed, with monitoring of hepatic function and triglycerides during prolonged therapy, based on its favorable association with CICU mortality compared to midazolam. In cases refractory to ketamine and propofol, we suggest using fentanyl infusion with bolus dosing of midazolam. In our experience, this combination is more hemodynamically neutral than dexmedetomidine. Dexmedetomidine use should be limited given its association with increased hypotension and bradycardia in the CICU (Central Illustration).^59^ In addition, antipsychotics or valproic acid can be prescribed as adjunctive sedatives, which necessitate vigilant monitoring for adverse effects, including QTc monitoring when using antipsychotics.

Regarding analgesia in CS, fentanyl analgosedation may also be appropriate in patients with a history of chronic pain/opioid use and relatively stable hemodynamics to mitigate pain-induced tachycardia and increased myocardial oxygen consumption.^120^ A nonopioid, non-NSAID regimen should be used in conjunction with systemic opioids to decrease overall opioid requirements. This strategy could mitigate the hypotensive effects of excessive opioid use. Furthermore, it can facilitate a shorter duration of mechanical ventilation and ICU length of stay.^96^

The management of analgesia and sedation in vasodilatory shock also requires a tailored approach based on the severity of organ dysfunction.^121^^,^^122^ There are limited data on the optimal anesthetic/sedative in a mechanically ventilated patient with sepsis, with a recent randomized controlled trial illustrating equipoise between dexmedetomidine and propofol regarding days without delirium and ventilator-free days (Table 1).^58^ In addition, a prospective analysis of septic shock patients compared anesthesia/sedation with propofol, midazolam, and dexmedetomidine and found no differences in HR, systolic blood pressure, and diastolic blood pressure; however, those sedated with propofol required increased time to awakening.^123^ Furthermore, when vasopressor utilization was retrospectively compared between those sedated with propofol vs midazolam, those in the propofol group required less norepinephrine at 6 hours and 24 hours than those in the midazolam group.^124^ However, patients with septic cardiomyopathy were not included in this trial, a population where sedation with dexmedetomidine may be deleterious. Similar to CS, a trial of ketamine as a preferred anesthetic/sedative may promote hemodynamic stability, with propofol as an alternative agent (Central Illustration). Dexmedetomidine may be appropriate if cardiac function is intact. Fentanyl and midazolam can be added for deeper sedation needs.

In mixed shock, to our knowledge, there are no current society guidelines or recent studies to guide optimal anesthetic and analgesic selection.^18^ Since mixed shock is a combination of cardiogenic and vasodilatory shock, a similar tailored approach to agent selection as outlined earlier can be considered.

As with shock in the CICU, there are limited data to guide anesthetic, sedative, and analgesic use in cases of severe HF.^119^^,^^125^ Extrapolating from the limited CS data, it may be preferable to use propofol over midazolam. An initial trial of ketamine may also be tenable in these patients, following the rationale already discussed. Dexmedetomidine has been observed to precipitate a larger decrease in MAP in those with LV dysfunction and has been associated with more hypotension, bradycardia, and inotrope use in the CICU.^59^^,^^126^ In patients with RV failure, using ketamine as an adjunct sedative to propofol and benzodiazepines has been described.^125^ In RV failure, decreasing chronotropy or inotropy can be even more deleterious than in LV failure, so we caution against the use of dexmedetomidine here. For analgesia in either LV or RV failure, a combined opioid and nonopioid, non-NSAID regimen is likely prudent.

Patients with malignant tachyarrhythmias, particularly electrical storm, often require deep sedation, and agents with sympatholytic mechanisms are beneficial.^119^ In addition to sympatholysis, propofol exerts antiarrhythmic effects in supraventricular and ventricular tachycardia.^127^^,^^128^ Propofol and midazolam are helpful agents in the treatment of electrical storm as they cause a chemical sympathectomy and provide anxiolysis which can help mitigate the sympathetic surge contributing to the malignant arrhythmia. For analgesia, opioids are likewise beneficial for tachyarrhythmias through sympatholysis with potential secondary antiarrhythmic effects.^129^ Systemic lidocaine infusion can offer analgesia in addition to rhythm control, mitigating excessive opioid use. Benzodiazepines are recommended as first-line sedation agents to provide anxiolysis and amnesia, while dexmedetomidine might have a potential role as a second-line agent to reduce sympathetic outflow.^130^ In refractory electrical storm, intubation may be required to allow for deep sedation. In these cases, the addition of propofol offers sympatholysis, antiarrhythmic effects, and deep sedation.

In patients admitted to the CICU post cardiac arrest, sedative selection can have important implications on neuroprognostication, particularly those who are undergoing targeted temperature management (TTM) after cardiac arrest. Plasma concentrations of drugs that undergo hepatic metabolism have been shown to be higher in TTM, likely due to reduced CYP450 activity.^131^ Accumulation of sedatives and analgesics during TTM can result in delayed awakening and can confound attempts to determine the extent of anoxic brain injury. In addition, TTM itself can cause hemodynamic alterations, with lower targeted temperatures being associated with a decreased CO, lower HR, and increased systemic vascular tone.^132^ The choice of sedative agent can either augment or attenuate these effects. The use of propofol plus remifentanil has shown shorter duration of mechanical ventilation and fewer delayed awakenings compared to the use of midazolam plus fentanyl, though the propofol-based regimen showed a greater need for vasopressors.^133^^,^^134^ Among CICU patients requiring deep sedation during TTM, propofol may be preferred to midazolam for earlier awakening. If unacceptable hypotension occurs, midazolam should be considered. Ongoing trials may help determine how to adjust sedation regimens in TTM.^135^

ETI is associated with dynamic hemodynamic changes.^136^ Common agents used during intubation are ketamine, propofol, midazolam, and fentanyl. Ketamine and etomidate are used to prioritize hemodynamic stability but may be complicated by postinduction tachycardia and a possible association with increased mortality, respectively. A meta-analysis of seven randomized clinical trials reported postintubation hypotension in up to 30% of patients receiving ketamine for RSI, however with lower vasopressor requirements and reduced adrenal suppression compared to etomidate.^70^ Of note, this meta-analysis included general intensive care patients and may not directly translate to the CICU population. Recently, a combination of ketamine and propofol, termed “ketofol,” has been used. Randomized controlled trials have shown that compared to etomidate, ketofol did not decrease MAP in the immediate postintubation period and was associated with significantly less adrenal insufficiency (Table 1).^57^ Compared to propofol alone, ketofol was associated with improved hemodynamic stability post intubation.^137^ However, this observation is limited by nonblinded drug administration. In the CICU, ketofol may be a viable choice promoting hemodynamic stability in RSI of patients with tenuous shock physiology at high risk of hemodynamic collapse during intubation. We suggest choosing induction agents for RSI tailored to the patient’s risk of hemodynamic collapse with intubation (Figure 1). Lastly, although neuromuscular blocking agents are not within the scope of this review, it is important to note that first-generation neuromuscular blocking agents used in ETI were associated with transient hypotension through histamine release. This is not commonly observed with the use of contemporary medications, such as rocuronium, making it a preferred choice in patients with cardiovascular disease.^138^

*Clinical Pearl: In the CICU patient undergoing ETI by RSI, physicians should consider their induction agents based on whether the patient is at high risk of hemodynamic collapse with intubation. For high-risk patients, ketamine, low-dose etomidate, and ketofol are reasonable choices.*

## Limitations of current practice recommendations

There are currently no specific society guidelines for anesthetic, sedative, and analgesic selection in the CICU. Current practices are informed by expert opinion, limited data, and extrapolation from general ICU guidelines.^119^^,^^122^ Regarding the choice of sedative, available data would suggest that propofol and dexmedetomidine are recommended over benzodiazepines for non-CICU patients. This seems to be motivated by the prolonged mechanical ventilation and ICU length of stay associated with benzodiazepines in clinical trials of critically ill medical and surgical patients. For analgesia, analgesic administration prior to sedation is recommended. This pathway reduces sedative requirements, duration of mechanical ventilation, ICU length of stay, and pain intensity. In addition, nonopioid adjunct agents are recommended to reduce systemic opioid requirements.^5^ Notably, none of the randomized clinical trials included CICU patients. Therefore, these recommendations are not generalizable to critically ill patients with cardiovascular disease.

Thus, there are gaps in our knowledge of how anesthetics, sedatives, and analgesics should be administered to CICU patients. There are limited data on optimal agent selection in cardiogenic and vasodilatory shock, and to our knowledge, no data on how these agents should be used in mixed shock.^43^^,^^58^ In these shock phenotypes, we have very limited trial dataon how these agents affect hemodynamic stability and CICU outcomes. In addition, the hemodynamic effects of anesthetics, sedatives, and analgesics in the setting of mechanical circulatory support are not well characterized. As their usage increases, there is a heightened need for large prospective and retrospective studies to determine guidelines on how to best select and titrate anesthesia, sedation, and analgesia in this CICU population. This will inform CICU-based anesthesia, sedation, and analgesia current practice and hopefully improve patient outcomes.

## Future directions

Regarding emerging therapies, volatile anesthetics have shown promise as novel inhaled anesthetics in the critically ill. They have increased in utilization since 2020 secondary to the COVID-19 pandemic-associated shortages of intravenous anesthetics/sedatives. Halogenated agents like sevoflurane are fast-acting, allowing for rapid anesthesia. In comparison to midazolam and propofol, they have been shown to reduce the duration of mechanical ventilation without associated worsening hemodynamic instability.^139^ However, halogenated agents are associated with dose-dependent reversible increases in intracranial pressure but are otherwise generally well tolerated.^140^^,^^141^ There are limited data for the use of volatile anesthetics in CS, though initial studies are promising. In patients with CS requiring veno-arterial extracorporeal membrane oxygenation, isoflurane was compared to standard intravenous sedatives including propofol, midazolam, and ketamine. Isoflurane was not associated with increased vasopressor requirements, nor increased extracorporeal membrane oxygenation flow rates compared to standard intravenous anesthetics/sedatives.^142^ Although more costly and requiring venting equipment to protect health care providers, inhaled volatile anesthetics could have a promising role in the CICU.

Precision medicine holds promise for tailoring anesthetic, sedative, and analgesic strategies to patient characteristics. Future studies, with robust outcome data, may enable the refinement of agent selection based on genetic profiles, biomarker levels, hemodynamic parameters, disease pathophysiology, or other phenotypic profiles, thereby further optimizing care in this vulnerable population.

## Conclusions

The management of anesthesia, sedation, and analgesia in the CICU requires a careful balance between ensuring patient comfort and preserving cardiac stability and overall patient safety. The complex interplay among preload, afterload (SVR), myocardial contractility, CO, HR, and respiratory changes poses unique challenges in patients with severe HF, CS, vasodilatory shock, or mixed shock phenotypes. Each anesthetic, sedative, and analgesic agent exhibits distinct hemodynamic and respiratory effects that must be carefully considered in clinical decision-making. Although propofol and dexmedetomidine offer rapid sedation and may reduce delirium, their risks of hypotension and bradycardia are significant. Ketamine, with its sympathomimetic effects, appears promising—especially when used in combination with propofol as “ketofol” during intubation—though its benefits may vary according to shock severity and the patient’s chronicity of illness. Etomidate’s neutral hemodynamic profile is offset by concerns about adrenal suppression and possible increased mortality. Opioids remain essential for analgesia; however, their potential for hypotension and respiratory depression necessitates the adjunctive use of nonopioid agents. In addition, when dosing anesthetics, sedatives, and analgesics, careful consideration must be given to changes in renal and hepatic metabolism.

The absence of CICU-specific guidelines underscores the important need for further research. Future efforts should focus on developing standardized, evidence-based protocols that integrate cardiovascular pathophysiology and, eventually, precision medicine approaches to improve outcomes for this high-risk population.

## Funding support and author disclosures

This work was supported in part by the Intramural Research Program of the 10.13039/100000002NIH. The views, information or content, and conclusions presented do not necessarily represent the official position or policy of, nor should any official endorsement be inferred on the part of, the Clinical Center, the National Institutes of Health, or the Department of Health and Human Services. Dr Washam is in the advisory board of Faraday Pharmaceuticals and receives research funding from Chiesi USA. Dr Alviar receives research grants from ACC and Baxter and is consulting for JNJ MedTexg and Zoll. All other authors have reported that they have no relationships relevant to the contents of this paper to disclose.
